# KBH-1, an herbal composition, improves hepatic steatosis and leptin resistance in high-fat diet-induced obese rats

**DOI:** 10.1186/s12906-016-1265-z

**Published:** 2016-09-13

**Authors:** Ji-Hye Lee, Jung-Jin Lee, Won-Kyung Cho, Nam-Hui Yim, Hyun-Kyu Kim, Bora Yun, Jin Yeul Ma

**Affiliations:** 1 Korean Medicine (KM) Application Center, Korea Institute of Oriental Medicine, Daegu, 700-300 Republic of Korea; 2 Nutraceutical Food R&D Center, Kolmar BNH, 22-15 Sandan-gil, Jeonui-myeon, Sejong 339-851 Republic of Korea

**Keywords:** Hepatic steatosis, Leptin resistance, *Saururus chinensis*, *Curcuma longa*, *Polygala tenuifolia*, KBH-1

## Abstract

**Background:**

KBH-1 is an herbal mixture of *Saururus chinensis, Curcuma longa* and *Polygala tenuifolia*. Each herb has been reported to have various pharmaceutical activities; however, the synergistic effect of this herbal composition on obesity has not yet been determined. We investigated the alleviation effect of KBH-1 and its possible molecular mechanism in obesity-induced hepatic steatosis and leptin resistance in the hypothalamus.

**Methods:**

We used HepG2 cells, primary neuronal cells and a high-fat diet (HFD)-induced obesity rat model to determine the effect of KBH-1 in vitro and in vivo on hepatic steatosis and leptin resistance accompanied by obesity. To identify the alleviation effect on lipid accumulation, HepG2 cells stimulated by FFA were stained with Oil Red O; in addition, immunoblotting and qPCR were performed to determine the effect of KBH-1 on the activation of proteins and nuclear enzymes in HepG2 cells and the steatotic liver of HFD-induced obesity rats. To examine the effect of KBH-1 on the leptin resistance of the hypothalamus and its possible molecular mechanism, we examined the effect of KBH-1 on the activation of the leptin resistance-related protein in primary cultured cortical neuron cells and the hypothalamus of an HFD-induced obesity rat model. In addition, we used HPLC analysis to identify the standard compound of KBH-1.

**Results:**

KBH-1 not only suppressed the lipid deposition in HepG2 cells exposed to free fatty acids (FFA) but also significantly down-regulated major factors in lipogenesis and up-regulated major factors in lipolysis. Similarly, in a HFD-induced obesity model, KBH-1 improved hepatic steatosis by alleviating the effects on lipogenic genes and kinases. In addition, KBH-1 significantly improved the leptin-mediated signals impaired by obesity or FFA in the obesity model and primary cultured cortical neuron cells. In addition, KBH-1 was analyzed to include six standard compounds using HPLC analysis, among these compounds, onji-saponin B and curcumin were potently suppressed the level of triglycerides.

**Conclusions:**

KBH-1 exhibits alleviating effects by improving hepatic steatosis and leptin resistance by up-regulating the activation of AMPK and suppressing the expression of PPARγ. These findings show the potential of KBH-1 as a functional food supplement or preventive agent in the treatment of obesity.

**Electronic supplementary material:**

The online version of this article (doi:10.1186/s12906-016-1265-z) contains supplementary material, which is available to authorized users.

## Background

Hepatic steatosis, a type of non-alcoholic fatty liver disease (NAFLD), is a general form of chronic liver disease and is caused by peripheral responses associated with obesity [1]. The main symptom of NAFLD is the accumulation of triglycerides (TG) and accompanying steatosis and obesity through leptin resistance as the pathological status in clinics [2]. Leptin, an adipose-derived hormone, is known to act on the hypothalamus of the brain to reduce food intake and increase energy spending by circulating at levels according to body fat mass [3]. Obesity enhances the leptin level by increasing adiposity and up-regulating enzyme expression, including β-oxidation and mitochondrial uncoupling, to dissipate the excess of energy. In previous studies, a diet-induced animal model is invariably accompanied by hyperleptinemia, and long-lasting hyperleptinemia causes the failure of appetite modulation by leptin resistance in the hypothalamus [4]. Finally, leptin has a critical role in modulating energy balance and anorexigenic hormones in the hypothalamus of the brain. Leptin acts by binding to a specific receptor, which has been identified as six isoforms of LepRa through LepRf [5]. Among the isoforms of leptin receptors, LepRb mediates most of the biological effects of leptin through the phosphorylation of both Janus kinase (JAK) 2 and signal transducer and activator of transcription (STAT) 3. The activation of JAK2 leads to the phosphorylation of tyrosine residues located in the intracellular domain of the LepRb, such as Tyr 985, Tyr 1077, and Tyr 1138 [6]. The phosphorylation of each tyrosine residue mediates the activation of extracellular signal-regulated kinase (ERK), STAT3, and STAT5, which leads to the increased transcription and expression of suppressors of cytokine signaling (SOCS) 3. SOCS3, which acts as an inhibitor by attenuating LepRb signaling, plays a central role in leptin resistance [6]. In addition, recent reports have suggested that leptin may act directly on the liver by influencing the leptin signaling and insulin signaling pathway and affecting hepatic lipid and lipoprotein metabolism [7]. Leptin acts mainly by activating the JAK3/STAT3 signaling in hepatic cells and results in the activation of phosphatidylinositol-3 kinase (PI3K)/AKT [8]. However, the pathway of 5-adenosine monophosphate-activated protein kinase (AMPK) and forkhead box protein (FOX) O1 was reported to be associated with leptin signaling, although their role in hepatic cells is not fully clear [9]. Therefore, the present study aimed to determine the modulation of leptin signaling molecule during the development of hepatic steatosis by a high-fat diet (HFD)-induced obesity model. In addition, the aim of this study is to identify the effect of KBH-1, standardized herbal composition, on steatosis and leptin resistance.

KBH-1, a novel herbal mixture, consists of Chinese lizard’s tail (*Saururus chinensis*)*,* turmeric (*Curcuma longa*) and Chinese senega (*Polygala tenuifolia*)*.* These herbs have been used as traditional Korean medicine in different countries, including China and southern Korea, for various purposes, and they have various pharmaceutical activities, such as anti-obesity effects, anti-inflammatory effects, anti-oxidative effects, neuroprotective effects, and the prevention of hypercholesterolemia [10–16]. *Saururus chinensis* has been used in folk medicine for the treatment of various inflammatory diseases, gonorrhea, and edema in Korea [17] and exhibits anti-asthmatic and anti-inflammatory activities [18]. In addition, previous biological studies of *Saururus chinensis* have established its effects in metabolic diseases, including hyperlipidemia, hyperglycemia, neuroprotective, and hepatoprotective effects [19–23]. *Curcuma longa* has been used in traditional medicine in China, Korea and India for ages as the main ingredient in prescriptions, such as Xiaoyao-san for mental disorders [24]. In addition, this extract has been used for the treatment of blood stasis in traditional Korean medicine [25]. Moreover, *Curcuma longa* is a main ingredient in Gambigyeongsinhwan, exerts an anti-obesity effect through lipid accumulation and adipose PPARα activation [26] and prevents high-fat diet-induced hyperlipidemia as a main ingredient in *Artemisia iwayomogi* [27]. *Polygala tenuifolia* has been used as a traditional Chinese medicine for the treatment of anxiety and dementia [28, 29], and the preventive effects of this extract on behavior disorders and inflammatory diseases have been demonstrated [30, 31].

The synergistic effect of *Saururus chinensis, Curcuma longa* and *Polygala tenuifolia* extracts on the complications that accompany obesity has not yet been determined. Therefore, as a preliminary study, we confirmed the efficacy of KBH-1 compared with each ingredient in the lipid accumulation of HepG2 cells (Additional file 1: Table S1). Therefore, in this study, the effects of KBH-1 in hepatic steatosis and leptin resistance in the hypothalamus were investigated, as was its possible molecular mechanism of action. Our results provide experimental evidence for the development of its use in supplementary foods.

## Methods

### Materials and reagents

*Polygala tenuifolia* Willdenow (China), *Curcuma longa* Linne (China) and *Saururus chinensis* Baill (Korea) were obtained from Yeongcheon Oriental Herbal Market (Yeongcheon, Korea). The identification of all plant materials was confirmed by Dr. Ki Hwan Bae of the College of Pharmacy, Chungnam National University (Daejeon, Korea). HepG2 cells were obtained from the American Type Culture Collection (Manassas, VA, USA). Oleic acid, palmitic aid, leptin, L-glutamine, polyethylenimine (PEI), D-glucose, and Oil Red O were obtained from Sigma Chemical (St. Louis, MO, USA). Fetal bovine serum, 100 U/ml penicillin/10 μg/ml streptomycin, Dulbecco's Modified Eagle Medium: Nutrient Mixture F-12 (DMEM/F12), Neurobasal Medium, B27, and L-glutamine were purchased from Gibco (Carlsbad, CA, USA). AdipoRed was obtained from Lonza (Walkersville, MD, USA). Hank’s Balanced Salt Solution (HBSS) was purchased from WELGENE (Gyeongsan-si, Korea). The anti-ERK1/2 (Thr202/Tyr204), phosphospecific ERK1/2 (p44/p42), anti-acetyl-CoA carboxylase (ACC), phosphospecific ACC (Ser79), phosphospecific STAT3 (Tyr 705), anti-AKT, phosphospecific AKT (Ser473), anti-AMPK, phosphospecific AMPK (Thr172), anti-RAS, phosphospecific c-Raf (Ser289/296/301), and anti-tubulin were obtained from Cell Signaling Technology, Inc. (Boston, MA, USA). The anti-leptin receptor was purchased from Abcam (Cambridge, UK). The anti-JAK2, phosphospecific JAK2 (Tyr1007/1008), and anti-beta-actin were obtained from Santa Cruz Biotechnology (Santa Cruz, CA). Secondary antibodies for immunoblot analysis, the ECL kit and the BCA protein assay kit were purchased from Thermo (Rockford, IL, USA). The PVDF membrane was purchased from Millipore (Darmstadt, Germany). The RNA extraction kit was obtained from Ambion (Austin, TX, USA). AccuPower RT PreMix, AccuPower GreenStar qPCR Master Mix and primers were purchased from Bioneer (Daejeon, Korea). Taqman gene expression assays and gene expression master mix were obtained from Applied Biosystems (ABI; NY, USA). The 10 % and 45 % kcal high-fat diets were purchased from Research Diet, Inc. (New Brunswick, USA). The green tea extract was purchased from Amax NutraSource, Inc. (Eugene, OR, USA). The leptin ELISA kit was purchased from MIoBs (Yokohama, Japan). The free fatty acid assay kit and thiobarbituric Acid Reactive Substances (TBARs) assay kit were purchased from Bioassay System (Hayward, CA, USA). The CCK-8 kit was obtained from Dojindo Molecular Technologies (Rockville, MD, USA). The GOT, GPT and TG, T-C assay kit were obtained from Asan Pharmaceutical (Seoul, Korea).

### Preparation of KBH-1

KBH-1 was composed of the same quantities of *Polygala tenuifolia, Curcuma longa Rhizoma* and *Saururus chinensis* as previously described [32]. Briefly, all voucher specimens were deposited in the herbal Bank of the KM-Based Herbal Drug Research Group, Korea Institute of Oriental Medicine. The three herb mixture were added to 60 % ethanol of ten times as primary extraction and five times as secondary extraction and then extracted by heating at 70-75 °C two times (3 h and 2 h) using COSMOS-660 (KYUNGSEO MACHINE Co, Incheon Korea). After extraction, the obtained KBH-1 extract was filtered out using Nylon 50 μm and concentrated using the Laborota 20 (Heidolph Ins., Germany) until 20-35 brix. The dextrin was added to the concentrate and prepared into powder by a spray dryer. The yield rate was 19 %.

### High performance liquid chromatography (HPLC) analysis

Using HPLC analysis, KBH-1 and its standard reference compounds, including quercitrin, onji-saponin B, bis-demethoxycurcumin (BDMC), demethoxycurcumin (DMC), curcumin and sauchinone, were standardized for quality control. Each standard stock was prepared by dissolving four standard compounds at 1 mg/mL in 100 % methanol. Sample stock solution was extracted at 10 mg/mL in 100 % methanol, which was sonicated at room temperature for 30 min. All standards and sample solution were filtered through a 0.22 μm syringe membrane filter from Whatman Ltd (Maidstone, UK) and stored at 4 °C before analysis. Chromatographic analysis of a reverse-phase HPLC system (Dionex Co., CA, USA) was performed with an ultimate diode array detector (DAD), injector 10 μL sample loop (Dionex, ID × L = 0.18 × 550 mm, Viper 550 mm, USA), ultimate 3000 pump and Chromeleon data acquisition system (Dionex, version 7, USA). Separation using an Optimapak C18 RP-column (250 × 4.6 mm, 5 μm, C_18_, Korea) was performed at 40 °C. The gradient elution was used with the following solvent systems: mobile phase A: double distilled water/triflouroacetic acid (TFA) (99.9/0.1; v/v), mobile phase B: acetonitrile. The run time of this system was 60 min, and the linear gradient method was applied to the mobile phase condition (solvent A: 90–30 %, B: 10–70 %, flow rate: 1.0 mL/min). The elution was performed with a gradient procedure as follows: 0–60 min, 2 % B; 2–60 min, from 2 % B to 98 % B.

### HepG2 cell culture and free fatty acid (FFA) treatment

Cells of the hepatocellular carcinoma cell line HepG2 were routinely cultured in DMEM/F12 supplemented with 10 % fetal bovine serum and 100 U/ml penicillin/streptomycin 10 μg/ml at 37 °C in a humidified atmosphere with 5 % CO_2_ in an incubator as previously described [33, 34]. The cells were grown to 75-80% confluence and then starved in serum-free medium overnight before treatment. For treatment experiments, the cells were treated with KBH-1 at 10, 30 and 90 μg/ml concentrations for 6 h and then exposed to a mixture of FFA (oleic acid/palmitic acid at 2:1) at a final concentration of 1 mM for 24 h. Fat droplets in cells were stained with Oil Red O dye. For Oil Red O staining, cells were fixed with 4 % neutral formaldehyde solution and stained with filtered Oil Red O solution for 15 min. After staining the lipid droplets, the staining solution was removed, and the plates were rinsed with water and photographs were taken using a Nikon digital camera system. To measure the intracellular triglyceride content, HepG2 cells were washed and incubated with the Adipored and then subjected to a Spectra Mas M2 fluorescence spectrophotometer (Molecular Devices, Sunnyvale, CA, USA) by the Adipored kit according to the manufacturer’s protocol. Cytotoxicity was tested using the Cell Counting Kit-8 according to the recommended methods.

### Primary neuronal cell culture and leptin resistance

Neuronal cells were primary cultured as described previously [35]. For primary cortical neuron cultures, the embryonic rat cortex was established from the 18-day embryos of SD rats (Samtako Bio Korea, Osan, Gyeonggido, Korea). Briefly, the cortex was removed and incubated for 15 min in HBSS containing 2 mg/ml trypsin. Cells were then dissociated by trituration and plated (5 × 10^6^ cells/ml) into PEI-coated plastic culture dishes containing Neurobasal Medium supplemented with 2 % B27, 0.5 mM L-glutamine, and 25 μM glutamate. Following cell attachment (24 h post-plating), the culture medium was replaced with Neurobasal Medium without glutamate. Experiments were performed using 7- to 9-day-old cultures. FFA can induce leptin resistance as described previously [36]. To detect leptin signaling, primary neurons were pretreated with 0.5 mM FFA (FFA;oleic acid:palmitic acid = 2:1) overnight prior to leptin treatment. For treatment studies, neurons were treated with KBH-1 for 1 h and then cotreated with 10 nM leptin as described previously [37]

### RNA isolation and RT-PCR

Total cellular RNA was extracted using TRIzol reagent according to the recommended methods. The quantity of isolated RNA was measured using a NanoDrop spectrophotometer (Thermo Scientific, Ltd., Waltham, MA) and processed for cDNA synthesis using the AccuPower RT PreMix cDNA synthesis kit according to the manufacturer’s protocol. Subsequently, SYBR Green-based qPCR amplification was performed using cDNA (extracted from HepG2 cells), 10 pmol of primers and AccuPower GreenStar qPCR Master Mix in the Applied QuantStudio 6 real-time PCR system (LifeScience, Carlsbad, CA USA) according to the instruction manual. The primer sequences for PCR analysis were as follows: CD36 (reverse) TTGATTTTGATAGATATGGGATGC, (forward) TGGAACAGAGGCTGACAACTT; SREBP1c (reverse) GGAAGGCTTCAAGAGAGGAGC, (forward) CGACATCGAAGACATGCTTCAG; SCD-1 (reverse) GCAGCCGAGCTTTGTAAGAGCGGT, (forward) CCTCTACTTGGAAGACGACATTCGC; ACC1 (reverse) TTCTGCTATCAGTCTGTCCAG, (forward) GCTGCTCGGATCACTAGTGAA; ACOX1 (reverse) CCACAGGACACCATTAAGC, (forward) GCGGACTACACTTCATAAATGC, CPT-1 (reverse) GGAGTGACCGTGAACTGAAAG, (forward) CCTCCGTAGCTGACTCGGTA; PPAR α (reverse) GGGAACAGATTTCCACATTG, (forward) TGGCTCTTGACCCTATTGG; beta-actin (reverse) TCGGCCACATTGTGAACTTT, (forward) TGGATCAGCAAGCAGGAGTA. Taqman probe–based qPCR amplification was performed using cDNA (extracted from animal organ), inventoried TaqMan gene expression assays and TaqMan Gene Expression Master Mix in the Applied QuantStudio 6 real-time PCR system according to the instruction manual. The inventoried probe assay ID for gene expression was as follows: Hmgcr, rCG44692 (Rn00565598_m1); Srebf1, rCG32702 (Rn01495769_m1); Cd36, rCG24451 (Rn01442639_m1); Actb, rCG42822 (Rn00667869_m1); Pparg, rCG29795 (Rn00440945_m1); Cpt1a, rCG48591 (Rn00580702_m1); Fasn, rCG33632 (Rn01463550_m1). The gene expression data were analyzed using the 2^-(ave.△△CT)^ method as described previously [38].

### Protein extraction and western blotting

Immunoblotting was performed as described previously [39, 40]. Samples were lysed in RIPA buffer consisting of 50 mM Tris-HCl (pH 8.0), 5 mM EDTA, 150 mM NaCl, 1 % NP-40, 0.1 % SDS, 1 mM PMSF, protease-inhibitor cocktail tablet, and phosphatase-inhibitor cocktail tablet. Cell lysates were centrifuged at 13,000 rpm for 30 min at 4°C. The protein concentration was determined using a BCA Protein Assay Kit. Protein samples were mixed with sample buffer (100 mM Tris-HCl [pH 7.6], 2 % SDS, 1 % 2-mercaptoethanol, 2 % glycerol, and 0.01 % bromophenol blue), incubated at 97 °C for 5 min. Approximately 15 μg of protein extract was loaded onto 4-20 % polyacrylamide gels. Electrophoresis was performed using a Mini Protein 3 Cell kit (Bio-Rad, Hercules, CA, USA). Resolved proteins were transferred onto a PVDF membrane. The membrane was first incubated in blocking buffer (10 mM Tris-HCl [pH 7.5], 150 mM NaCl, 0.1 % Tween 20, and 3 % BSA) and then incubated overnight at 4°C with 1000 diluted primary antibodies. After washing with a washing buffer (10 mM Tris-HCl [pH 7.5], 150 mM NaCl, and 0.1 % Tween 20) three times for 20 min each, the membrane was probed with 5000 diluted secondary antibody for 1 h at room temperature. The membrane was then washed with washing buffer three times for 10 min each and developed using an ECL kit. Chemiluminescent signals were detected using a LAS-4000 Luminescent Image Analyzer (Fuji Photo Film Co., Japan). Band intensities were quantified using ImageJ software (National Institutes of Health, USA).

### High-fat diet (HFD)-induced obesity rat model

All animal experiments were performed according to the Guide for the Care and Use of Laboratory Animals of the National Institutes of Health (NIH publication No. 83-23, revised 1996) and were approved by the Institutional Animal Care and Use Committee of KIOM (Approval No. 14-027). Male SD rats were purchased from Samtako Co. (Osan, Korea). All animals were housed in a room with controlled temperature (20-24 °C), humidity (40-60 %) and lighting (12 h light/dark cycle) and were supplied with water *ad libitum*. After acclimation for 1 week, animals were randomly divided into 2 groups: the normal group was fed with a 10 % kcal fat diet (ND), whereas the obesity model group was fed with a 45 % kcal fat diet (HFD). After 7 weeks of feeding with the HFD, the resultant obese rats in the HFD group were subdivided into 4 groups: (1) HFD: 45 % kcal fat diet-fed rats; (2) PC: HFD treated with 200 mg/kg body weight/day of green tea extract (PC) administration; (3) KBH-1 150: HFD treated with 150 mg/kg body weight/day of KBH-1 extract administration; and (4) KBH-1 300: HFD treated with 300 mg/kg body weight/day of KBH-1 extract administration. Then, drugs were orally administered as a suspension in saline for 8 weeks with a HFD diet (KBH-1 150 and KBH 300 groups). The same amount of saline was orally administered to the ND and HFD groups for 8 weeks with feeding ND or HFD diet (ND and HFD groups). At the end of the experimental period, animals were anesthetized by CO_2_ gas, blood was collected, and organs were excised. The organs were rinsed with saline solution, weighed and stored at -80 °C.

### Measurement of biochemical parameters and oxidative stress

Serum total cholesterol (T-C), low density lipoprotein-cholesterol (LDL-C), high density lipoprotein-cholesterol (HDL-C), triglyceride (TG), glucose, glutamic-oxaloacetic transaminase (GOT), glutamic pyruvic transaminase (GPT), lactate dehydrogenase (LDH), UREA, creatinine and alkaline phosphatase (ALP) levels were measured using an Erba XL-200 automated clinical chemistry analyzer (Mannheim, Germany). Serum leptin, TNFα and IL-1β levels were measured with a commercial ELISA kit according to the manufacturer’s protocol. Markers of oxidative stress, such as malondialdehyde (MDA), were measured with commercial TBARs assay kit according to the manufacturer’s instructions.

### Hepatic lipid extraction and measurement

Hepatic lipid extraction was performed as previously described [39]. Wet livers (100 mg each) were homogenized in 0.5 ml of 1 M NaCl. For lipid extraction, 3 ml of chloroform/methanol (2:1) was added to 0.5 ml of liver tissue homogenate and vortexed for 2 h. Then, the mixture was centrifuged at low speed, and the lipid-containing phase was collected, dried and re-suspended in 0.5 ml of Triton X-100/methanol (2:1). TG and T-C were measured with a commercial assay kit according to the manufacturer's protocol.

### Histological analysis

Gonadal adipose and liver tissues were fixed in 4 % neutral formaldehyde solution. Adipose tissue and liver tissue were subsequently dehydrated in a graded ethanol series (70-100 %) and embedded in paraffin. The tissues were sectioned (4 μm thick) using a Leica RM 2165 rotary microtome (Wetzlar, Germany) and stained with hematoxylin and eosin (H&E). Sections were viewed with an Axioskop 40 (Oberkochen, Germany) and photographed at 100× magnification.

### Statistical analysis

The data are presented as the mean ± the standard error of the mean (SEM) and analyzed by GraphPad Prism software. An unpaired one-way ANOVA was used as indicated, followed by Dunnet’s test to ascertain statistical significance.

## Results

### KBH-1 alleviates free fatty acid-stimulated lipid deposition and the activation of lipogenic enzymes in HepG2 cells

**Fig. 1:**
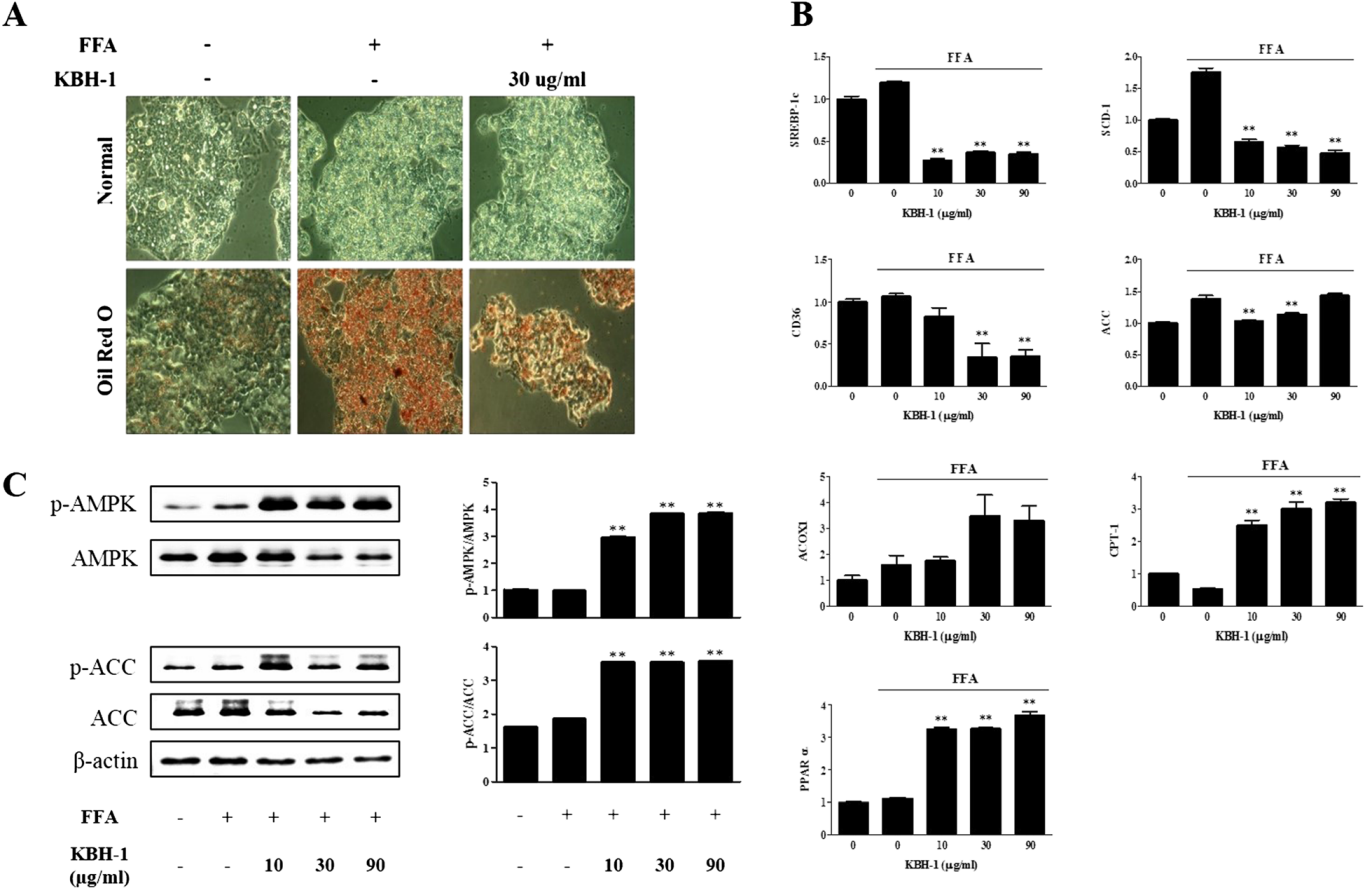
Effect of KBH-1 on lipid accumulation and the activation of lipogenic enzymes in HepG2 cells. HepG2 cells cultured in DMEM/F12 supplemented with 10% fetal bovine serum, then exposed to a mixture for FFA (oleic acid/palmitic acid at 2:1) at a final concentration of 1 mM for 24 h, and the effect of various concentrations of KBH-1 (10–90 μg/ml) was monitored. (**a**) Fat droplets in cells were stained with Oil Red O dye. (**b**) The FFA-induced phosphorylation of AMPK and ACC was measured using SDS-PAGE and immunoblotting. ACC, AMPK, and β-actin were used for normalization (n = 3). (**c**) The FFA-induced mRNA expression of major lipogenic transcription factors (SREBP-1c, SCD-1, CD36, ACC, ACOX1, CPT-1 and PPARα) was analyzed by quantitative real-time PCR. The results are expressed as a relative density after normalization to the β-actin mRNA level. The data are expressed as the mean ± SEM. Significant differences from each control (no KBH-1 treatment) are indicated by **p < 0.001

To investigate the alleviation effect of KBH-1 extract on the lipid accumulation of HepG2 cells, Oil Red O staining was performed with KBH-1 at a concentration of 30 μg/ml decreased lipid accumulation in HepG2 stimulated by FFA (Fig. 1a). Therefore, to identify the effect of KBH-1 on the activation of proteins and nuclear enzymes of HepG2 cells exposed to FFA, we examined the mRNA level of sterol regulatory element-binding protein (SREBP)-1c, stearoyl-CoA (SCD)-1, CD36, acetyl-CoA carboxylase (ACC), acyl-CoA oxidase (ACOX) 1, carnitine palmitoyltransferase (CPT)-1 and peroxisome proliferator-activated receptor (PPAR) α. As shown in Fig. 1b, the expression level of SREBP-1c, SCD-1, CD36 and ACC, as a major factors in lipogenesis, significantly down-regulated by treatment with KBH-1, whereas, the expression level of ACOX1, CPT-1 and PPARα, as a major factors in lipolysis, was elevated in a dose-dependent manner. Similarly, the phosphorylation of adenosine monophosphate-activated protein kinase (AMPK) and ACC proteins also increased after treatment with KBH-1 (Fig. 1c). These results indicate that KBH-1 improves fatty acid catabolism at the transcriptional level by regulating the expression of lipogenic genes and proteins.

### KBH-1 reduces hepatic steatosis in an HFD-induced obesity model

**Fig. 2:**
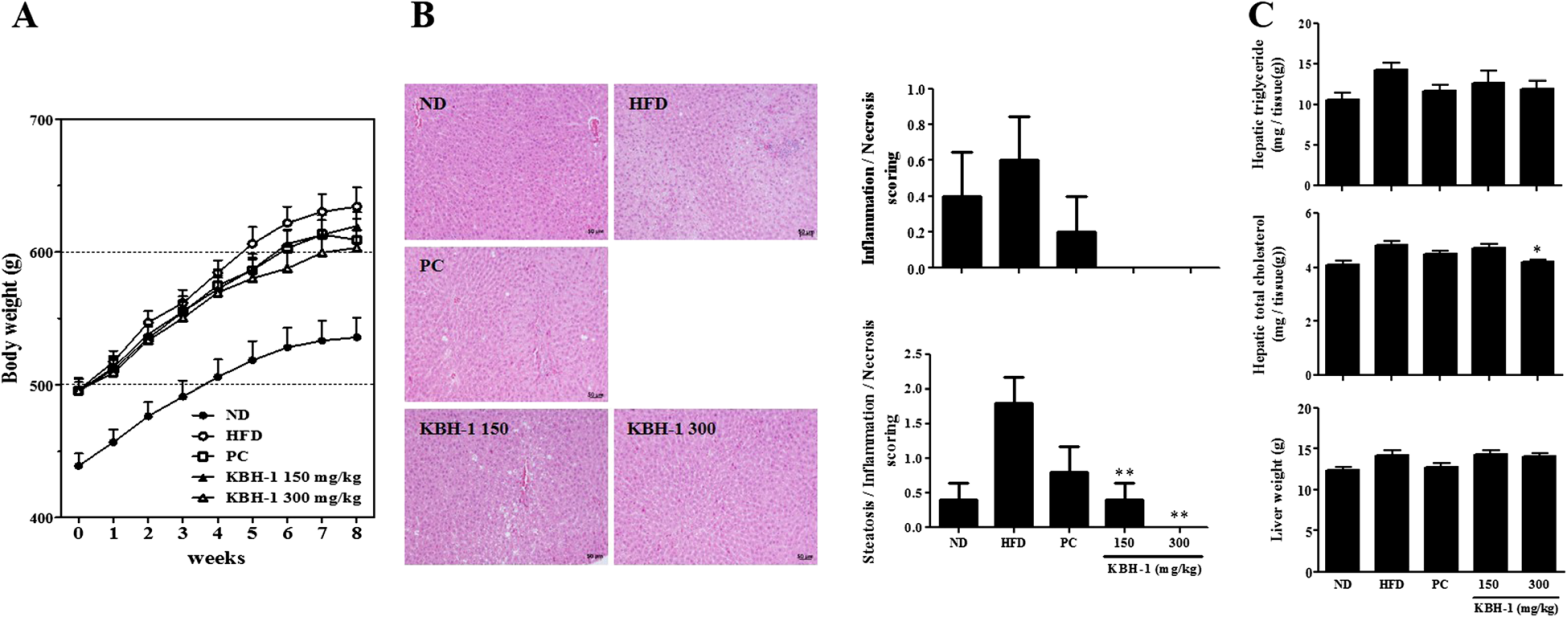
Effect of KBH-1 on hepatic steatosis of the HFD-induced obesity model. Animals were subdivided into 5 groups: ND, HFD, PC (treated with 200 mg/kg of green tea extract), KBH-1 150 mg/kg, and KBH-1 300 mg/kg. (**a**) The body weight change of each group in an HFD-induced obesity model. (**b**) Representative hematoxylin-eosin staining. (**c**) The changes of hepatic TG and TC and liver weight. The data are expressed as the mean ± SEM. Significant differences from the HFD group are indicated by *p < 0.05 or **p < 0.001

We investigated the effect of KBH-1 on hepatic steatosis. First, we analyzed the body weight (Fig. 2a) and body weight gain (Additional file 2: Figure S2) of HFD-induced rats. Rats in the KBH-1 group exhibited a significant decrease in body weight and body weight gain. HFD-induced rats exhibited a significant increase in the level of GPT and LDL-C, and KBH-1 suppressed the levels of GPT, LDL-C, and TG (Additional file 3: Figure S1). Although the extent of hepatocytes with visible lipid accumulation weakly caused HFD-induced hepatic steatosis, and KBH-1 at a concentration of 150 and 300 mg/kg significantly improved hepatic steatosis, including steatosis scoring and hepatic TC (Fig. 2b-c).

### KBH-1 suppressed lipogenesis in hepatic steatosis

**Fig. 3:**
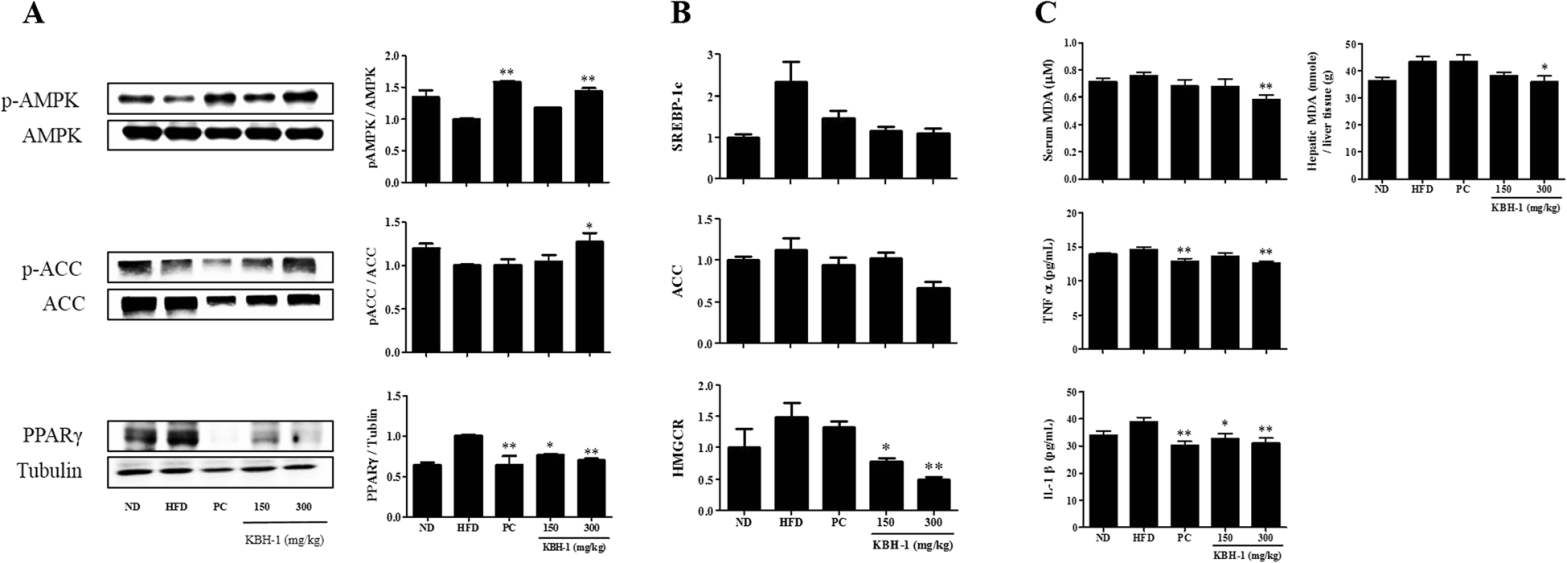
Anti-lipogenic effect of KBH-1 on hepatic steatosis. (**a**) Liver tissue was homogenized and the lysates were subjected to western blotting for AMPK, ACC phosphorylation and PPARγ expression. (**b**). Liver tissue was homogenized and SREBP-1c, ACC and HMGCR were analyzed by quantitative real-time PCR. (**c**) Serum and hepatic MDA, TNFα and IL-1 β was measured with a commercial TBAR assay kit and ELISA kit. The data are expressed as the mean ± SEM. Significant differences from the HFD group are indicated by *p < 0.05 or **p < 0.001

To identify the molecular mechanism with regard to the inhibitory effect of KBH-1 on hepatic steatosis, we investigated the effect on the activation of AMPK, ACC and PPARγ proteins or the expression of SREBP-1c, ACC and 3-hydroxy-3-methylglutaryl CoA reductase (HMGCR) genes. As shown in Fig. 3a, KBH-1 not only up-regulated the phosphorylation level of AMPK and ACC, as the kinase inhibited hepatic lipogenesis, but also inhibited the expression of PPARγ protein associated with the increased expression of lipogenic genes and hepatic TG accumulation [41, 42]. Moreover, KBH-1 concentration-dependently suppressed the expression of SREBP-1c, ACC and HMGCR genes as a major factor in adipogenesis, which promotes fat accumulation in the liver [43] (Fig. 3b). In addition, to determine the effect of KBH-1 on lipid peroxidation and inflammatory cytokines in the steatotic liver, we assessed serum and hepatic malondialdehyde (MDA), tumor necrosis factor (TNF)-α, and IL-1β. As a result, KBH-1 significantly inhibited serum and hepatic MDA, whereas green tea extract, as a positive control, had no direct effect. KBH-1 also dose-dependently suppressed the TNF-α and IL-1β released from the steatotic liver.

### KBH-1 improves the leptin resistance of the hypothalamus in the HFD-induced obesity model

**Fig. 4:**
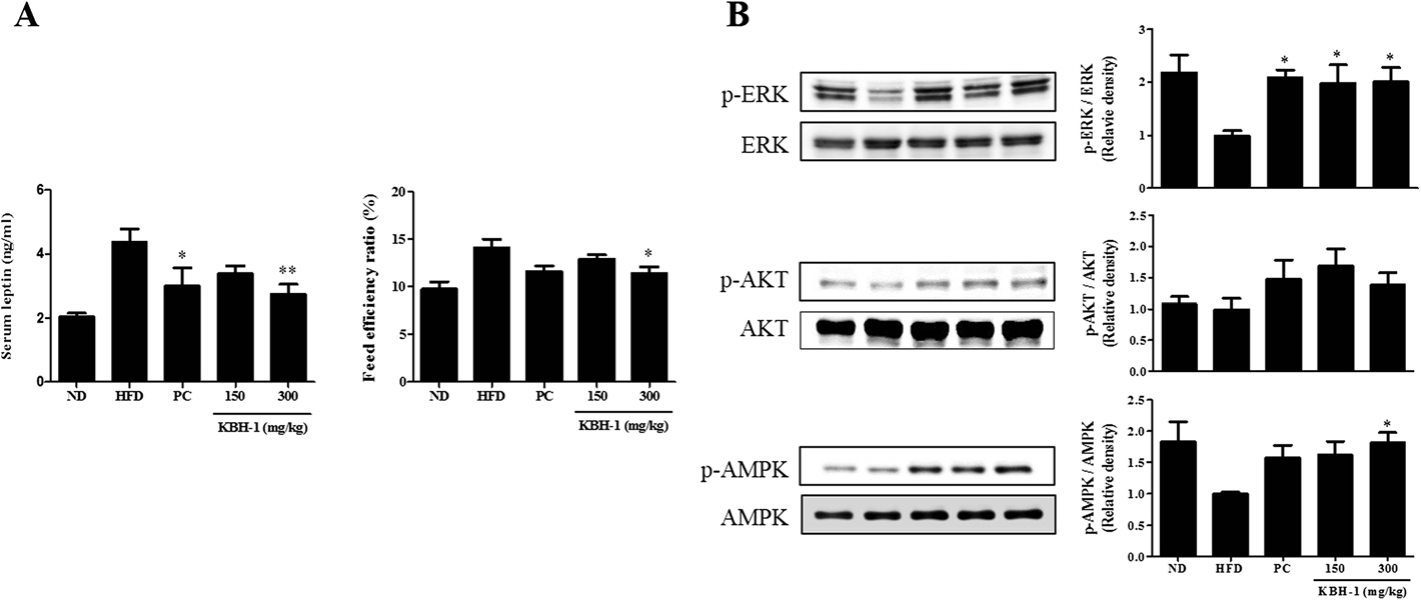
Effect of KBH-1 on the leptin resistance of the hypothalamus. (**a**) Hypothalamus tissue was homogenized and (A) serum leptin was measured with an ELISA kit. The feed efficiency ratio (%) was calculated as weight gain/food intake × 100. (**b**) The phosphorylation of ERK, AKT and AMPK was measured using western blotting. The data are expressed as the mean ± SEM. Significant differences from the HFD group are indicated by *p < 0.05 or **p < 0.001

Next, we investigated the effect of KBH-1 on the leptin resistance of the hypothalamus and its possible molecular mechanism. First, in HFD-induced obesity rats, we assessed the serum leptin level and the feed efficiency ratio. KBH-1 suppressed the serum leptin level of the HFD group increased in comparison with the normal diet (ND) group; similarly, the feed efficiency ratio was decreased (Fig. 4a). Therefore, we investigated the effect of KBH-1 on the phosphorylation of ERK, AKT and AMPK was down-regulated by leptin resistance in hypothalamus. KBH-1 significantly activated the phosphorylation of ERK and AMPK in a concentration-dependent manner, whereas AKT phosphorylation was non-significantly activated (Fig. 4b).

### KBH-1 alleviates the resistance of leptin-mediated signaling in primary cultured cortical neuron cells

**Fig. 5:**
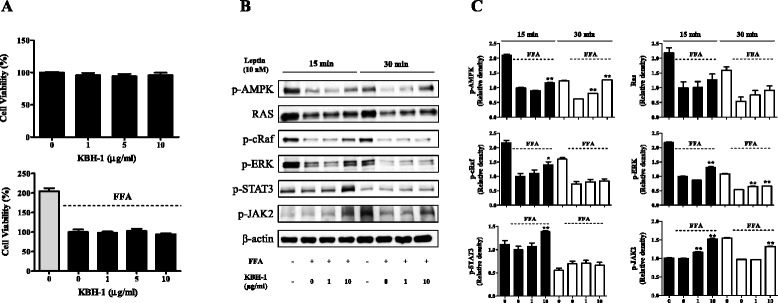
Effect of KBH-1 on leptin-resistant cortical neuron cells. Cells were cultured in neurobasal medium supplemented with 2% B27, 0.5 mM L-glutamine, and 25 μM glutamate. After 24 h, the culture medium was replaced with neurobasal medium without glutamate. For the resistance experiments, primary neurons were pretreated with 0.5 mM FFA (oleic acid:palmitic acid = 2:1) overnight prior to leptin treatment. For treatment studies, neurons were treated with KBH-1 for 1 h, then treated with 10 nM leptin. (**a**) Cell viability was determined by the CCK assay. (**b**) The activation of AMPK, RAS, cRaf, ERK, STAT3 and JAK2 were measured using SDS-PAGE and immunoblotting. β-actin was used for normalization (**c**, n = 3). The data are expressed as the mean ± SEM. Significant differences from each control (no KBH-1 treatment) are indicated by *p < 0.05 or **p < 0.001

In primary cultured cortical neuron cells, we confirmed the alleviated effect of KBH-1 on leptin resistance. In leptin-resistant neurons induced by FFA, the activation of leptin-mediated signals, such as AMPK, Ras, cRaf, ERK, STAT3 and JAK2, was down-regulated. KBH-1 significantly improved the phosphorylation of AMPK, cRaf, ERK, STAT3 and JAK2 in cortical neuron cells treated with leptin for 15 min (Fig. 5b). Although the alleviation effect of KBH-1 in cortical neuron cells treated with leptin for 30 min was weak, KBH-1 significantly up-regulated the phosphorylation of AMPK, Raf, ERK and JAK2. These findings indicate that KBH-1 can be used to alleviate the leptin resistance of primary cultured cortical neuron cells in a manner independent of cytotoxicity (Fig. 5a).

### Standardization of KBH-1 and its standard compounds

**Fig. 6:**
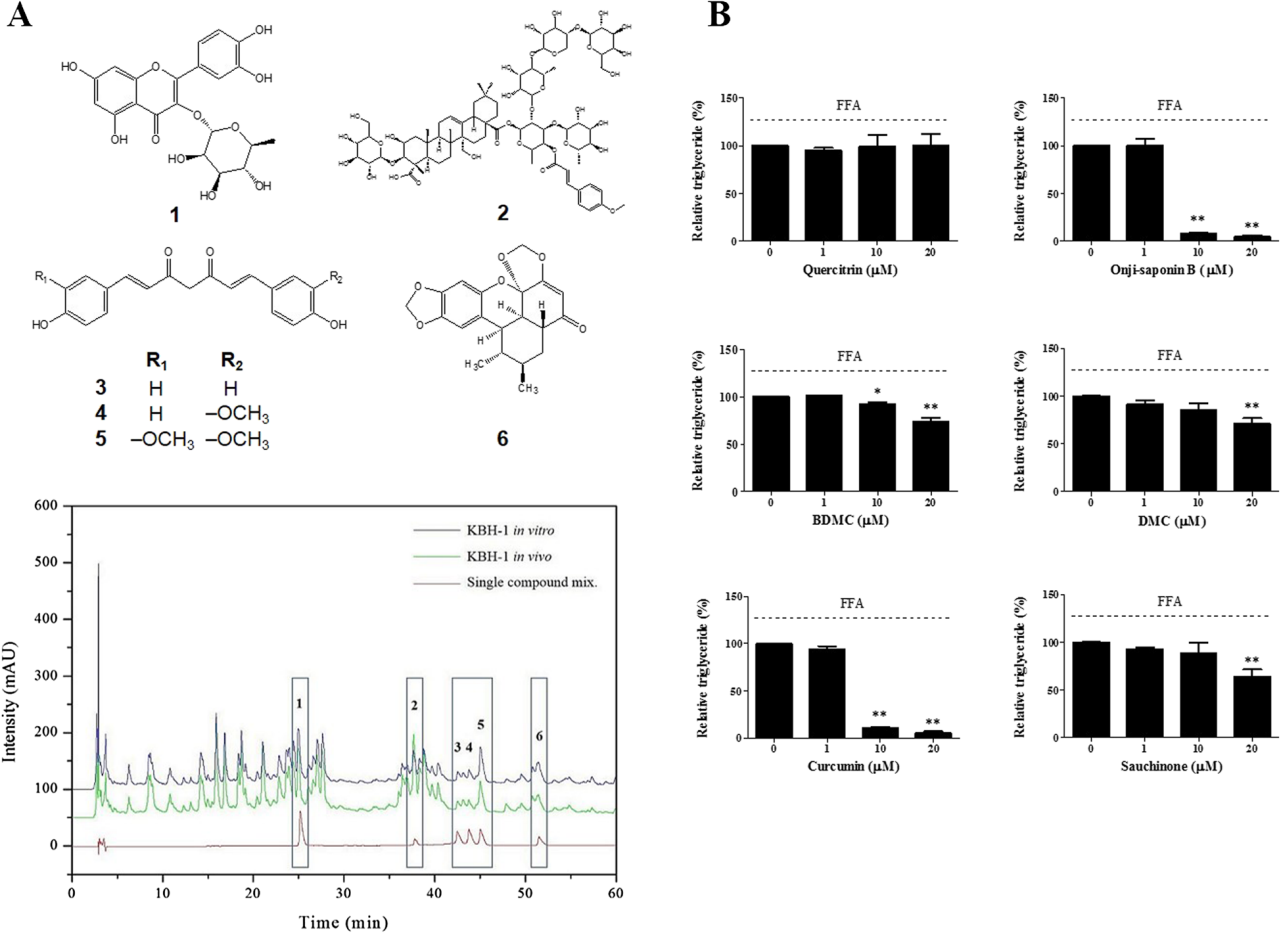
HPLC profile of KBH-1 and identification of active compound. The HPLC chromatogram of the six standard compounds was monitored at 280 nm. Black, green and red lines represent KBH-1 used in vitro, KBH-1 used in vivo with diluting agent and a mixture of six single compounds (**a**). HepG2 cells cultured in DMEM/F12 supplemented with 10% fetal bovine serum, then exposed to a mixture for FFA (oleic acid/palmitic acid at 2:1) at a final concentration of 1 mM for 24 h, and the effect of various concentrations (1-20 μg/ml) of six standard compounds (quercitrin, onji-saponin B, BDMC, DMC, curcumin and sauchinone) was monitored. (**b**) The TG level in cells was stained with Adipore. The data are expressed as the mean ± SEM. Significant differences from each control (no KBH-1 treatment) are indicated by *p < 0.05 or **p < 0.001

In HPLC analysis, KBH-1 was analyzed to include six standard compounds: quercitrin, onji-saponin B, BDMC, DMC, curcumin and sauchinone (Fig. 6a). Thus, to determine the mechanism of activation, we examined the level of triglycerides (TGs) in HepG2 cells. The results showed that onji-saponin B, BDMC, DMC, curcumin and sauchinone significantly decreased TG levels; among these active compounds, onji-saponin B and curcumin were potently suppressed at a concentration greater than 10 μM (Fig. 6b).

## Discussion

The present study shows the effect of KBH-1 in the treatment of steatosis and liver and accompanying leptin resistance in the hypothalamus through continuous feeding with a HFD for up to 15 weeks. We showed that the HFD group increased the lipid accumulation in liver and lowered the sensitivity of leptin-mediated signals in the hypothalamus in comparison with those fed with ND. We demonstrated that KBH-1, as an herbal composition of *Polygala tenuifolia, Curcuma longa,* and *Saururus chinensis* extracts, improved hepatic steatosis and leptin resistance in a HFD-induced obesity model through the increase of AMPK phosphorylation and the up-regulation of leptin-mediated signals. Although further studies, including clinical trials, are required, KBH-1 may be used as a functional food supplement to manage obesity. Presently, KBH-1 is examining the clinical trials based on no observable adverse effect level resulted from the subacute toxicity test.

NAFLD is considered to be the insulin resistance or the hepatic component of metabolic syndrome, as it increases with obesity and type 2 diabetes mellitus [44]. In previous studies, obesity was shown to be associated with higher levels of leptin in serum and hepatic steatosis after lipid accumulation [45]. Therefore, our study shows that KBH-1 inhibited lipid accumulation by up-regulating AMPK phosphorylation; moreover, we determined the effect of the major factor genes in lipogenesis and lipolysis *in vitro* (Fig. 1). Our results showed that KBH-1 suppressed the mRNA expression of SREBP-1c, SCD-1, CD36 and ACC and increased the mRNA expression of ACOX1, CPT-1 and PPARα, which are the primary enzymes in lipogenesis. These results indicated that the anti-lipogenic effect of KBH-1 occurred at the transcriptional level, as SREBP-1 and PPARα, regulators of lipogenesis and lipolysis, respectively, positively modulate lipid metabolism by inducing the expression of target genes that encode rate-limiting enzymes, such as ACC, and up-regulating the expression of the genes involved in the transport and oxidation of fatty acids, such as CPT-1 and CD36 [46]. Similarly, in an in vivo hepatic steatosis model caused by obesity, KBH-1 not only suppressed the increase of body weight and body weight gain but also inhibited the increased the steatosis score and level of hepatic TC (Fig. 2). In lipid metabolism, AMPK, as a key regulator in lipid metabolism, has been well-known to modulate cholesterol synthesis, hepatic fatty acids, and fatty acid oxidation [47]. Importantly, AMPK phosphorylation suppresses major factors in lipogenesis, such as SREBP-1 and HMGCR [48]. KBH-1 significantly increased the AMPK protein inhibited by HFD in liver and also elevated ACC expression, which is a marker of AMPK activation and a modulator in the synthesis and oxidation of fatty acids (Fig. 3) [49]. KBH-1 significantly suppressed the phosphorylation of PPARγ, which is a transcription factor of TG synthesis and the master regulator of adipogenesis [50]. In accordance with these results, the mRNA expression of SREBP-1, ACC and HMGCR was suppressed by treatment with KBH-1 (Fig. 3). In addition, KBH-1 significantly inhibited the serum and hepatic levels of MDA, TNFα, and IL-1β, the last of which is a lipid peroxidation factor and inflammatory cytokines released from steatotic liver (Fig. 3) [51].

Obesity causes leptin resistance in the hypothalamus accompanied by hepatic steatosis in the liver. Leptin resistance down-regulated the phosphorylation of the leptin-mediated signals, such as ERK, AKT, and AMPK [52]. When leptin resistance in the hypothalamus was caused by obesity, the level of serum leptin and the feed efficiency ratio was increased [53]. KBH-1 not only suppressed the leptin level and feed efficiency ratio but also enhanced the activation of ERK, AKT and AMPK proteins. These results show that KBH-1 may be used to improve the leptin resistance in the hypothalamus caused by obesity (Fig. 4). Therefore, in addition, we identified the possible mechanism of KBH-1 in primary cultured cortical neuron cells pre-treated with FFA in vitro.

In accordance with the results of HFD-induced obesity in vivo, KBH-1 significantly up-regulated the activation of leptin-mediated signals, including AMPK and JAK2, in a time-dependent manner (Fig. 5b). These results indicate that KBH-1 can be used to alleviate the leptin resistance of cortical neuron cells, which was not due to cytotoxicity (Fig. 5a).

In addition, to standardize KBH-1, we detected six standard compounds, including quercitrin, onji-saponin B, BDMC, DMC, curcumin and sauchinone, using HPLC analysis (Fig. 6a). Next, to identify the active compound of KBH-1, we investigated the level of TG in hepG2 stimulated by FFA. As a result, onji-saponin B and curcumin potently suppressed the level of TG stimulated by FFA (Fig. 6b).

## Conclusion

We have obtained experimental evidence that KBH-1, including onji-saponin B and curcumin, improves the hepatic steatosis and leptin resistance of hypothalamus in an HFD-induced obesity rat model by up-regulating the activation of AMPK and suppressing the expression of PPARγ in the liver and enhancing the leptin-mediated signals in the hypothalamus. Therefore, we judiciously expect that KBH-1 extract may be used as a functional food supplement or as a preventive agent to manage the complication of obesity.

## Additional file

Additional file 1: Table S1. Blood chemical analysis on high-fat diet (HFD)-induced obesity rat model . HFDinduced rats show significant increase the level of GPT and LDL-C, and KBH-1 suppressed the level of GPT, LDL-C, and TG. (DOCX 16 kb) Additional file 2: Figure S2. Effect of KBH-1 on hepatic steatosis of HFD-induced obesity model. Animals were subdivided into 5 groups: ND, HFD, PC (treated with 200mg/kg of green tea extract), KBH-1 150mg/kg, and KBH-1 300 mg/kg. The body weight change of each group on HFD-induced obesity model. Data are expressed as the mean ± SEM. Significant differences from HFD group are indicated by *p < 0.05, **p < 0.01, or ***p < 0.005. (DOCX 55 kb) Additional file 3: Figure S1. Effect of KBH-1 on cell viability and lipid accumulation. HepG2 cells was routinely cultured in DMEM/F12. The cells were treated with various concentration (0-90 μg/ml) of Saururus chinensis (SC), Curcuma longa (CL), Polygala tenuifolia (PT) or KBH-1 extracts for 6 h, then exposed to a mixture for FFA (oleic acid/palmitic acid at 2:1). (A) Intracellular triglyceride contents were examined using the AdipoRed Assasy kit. (B) Cell viability was determined by the CCK assay. The results were confirmed by three independent experiments, which were each conducted in triplicate. Data are expressed as the mean ± SEM. Significant differences from control (0 μg/ml) are indicated by *p < 0.05, or ***p < 0.005. (DOCX 42 kb)

## Abbreviations

ACC: Anti-acetyl-CoA carboxylase
ACOX: Acyl-CoA oxidase
ALP: Alkaline phosphatase
AMPK: 5-adenosine monophosphate-activated protein kinase
AUC: Area under the curve
CPT: Carnitine palmitoyl transferase
DMEM/F12: Dulbecco's Modified Eagle Medium:Nutrient Mixture F-12
ERK: Extracellular signal-regulated kinase
FFA: Free fatty acid
FOX: Forkhead box protein
GOT: Glutamic-oxaloacetic transaminase
GPT: Glutamic pyruvic transaminase
H&E: Hematoxylin and eosin
HDL-C: High density lipoprotein-cholesterol
HFD: High-fat diet
JAK: Janus kinase
LDH: Lactate dehydrogenase
LDL-C: Low density lipoprotein-cholesterol
MDA: Malondialdehyde
NAFLD: Non-alcoholic fatty liver disease
ND: Normal diet
OGTT: Oral glucose tolerance test
PEI: Polyethylenimine
PI3K: Phosphatidylinositol-3 kinase
PPAR: Peroxisome proliferator-activated receptor
SCD: Stearoyl-CoA
SEM: The standard error of the mean
SOCS: Suppressors of cytokine signaling
SREBP: Sterol regulatory element-binding protein
STAT: Signal transducer and activator of transcription
TBAR: Thiobarbituric acid reactive substances
T-C: Total cholesterol
TG: Triglyceride
TNF: Tumor necrosis factor
