# Supplementary material for: KBH-1, an herbal composition, improves hepatic steatosis and leptin resistance in high-fat diet-induced obese rats
Source: BMC Complement Altern Med. 2016 Sep 13;16(1):355. doi: 10.1186/s12906-016-1265-z (PMC5020448; doi:10.1186/s12906-016-1265-z)
Supplement: Additional file 1: Table S1. — Blood chemical analysis on high-fat diet (HFD)-induced obesity rat model . HFDinduced rats show significant increase the level of GPT and LDL-C, and KBH-1 suppressed the level of GPT, LDL-C, and TG. (DOCX 16 kb) [file 12906_2016_1265_MOESM1_ESM.docx]

Table S1. Blood chemical analysis on high-fat diet (HFD)-induced obesity rat model.

|  | **ND** | **HFD** | **PC** | **KBH-1 150** | **KBH-1 300** |
| --- | --- | --- | --- | --- | --- |
| Hepatic function(IU/L) | | | | | |
| **SGOT** | **133.13±6.26** | **155.75±6.67** | **160.38±10.37** | **146.38±5.70** | **141.38±3.21** |
| **SGPT** | **28.88±1.03***** | **38.13±1.75** | **35.00±1.63** | **36.13±1.74** | **31.50±1.40*** |
| **ALP** | **67.25±2.72** | **66.75±1.52** | **78.50±3.30** | **76.38±4.31** | **79.63±3.36** |
| Renal function(mg/dL) | | | | | |
| **UREA** | **14.46±0.68** | **14.73±0.37** | **15.58±0.51** | **14.26±0.30** | **14.51±0.47** |
| **CREATININE** | **0.68±0.03** | **0.76±0.02** | **0.80±0.04** | **0.76±0.03** | **0.81±0.05** |
| Lipid parameters in blood (mg/dL) | | | | | |
| **TRIGLYCERIDE** | **49.50±6.07** | **46.88±2.71** | **45.00±3.68** | **44.50±2.46** | **34.00±1.75**** |
| **CHOLESTEROL** | **61.25±2.94** | **66.38±3.17** | **58.25±2.97** | **61.13±2.82** | **60.25±2.50** |
| **High Density Cholesterol** | **44.88±1.89** | **46.13±1.75** | **44.88±1.37** | **44.38±0.96** | **43.88±1.19** |
| **Low Density Cholesterl** | **13.48±0.67**** | **17.69±0.54** | **15.60±0.57** | **16.29±0.68** | **15.27±0.44**** |
| **Free fatty acid** | **437.4±24.73** | **478.3±20.42** | **473.2±10.89** | **456.9±41.77** | **423.7±11.32** |

Values are expressed as the mean ± SEM. Significant differences from HFD group are indicated by **p* < 0.05 or ***p* < 0.001.
