# Supplementary material for: KBH-1, an herbal composition, improves hepatic steatosis and leptin resistance in high-fat diet-induced obese rats
Source: BMC Complement Altern Med. 2016 Sep 13;16(1):355. doi: 10.1186/s12906-016-1265-z (PMC5020448; doi:10.1186/s12906-016-1265-z)
Supplement: Additional file 2: Figure S2. — Effect of KBH-1 on hepatic steatosis of HFD-induced obesity model. Animals were subdivided into 5 groups: ND, HFD, PC (treated with 200mg/kg of green tea extract), KBH-1 150mg/kg, and KBH-1 300 mg/kg. The body weight change of each group on HFD-induced obesity model. Data are expressed as the mean ± SEM. Significant differences from HFD group are indicated by *p < 0.05, **p < 0.01, or ***p < 0.005. (DOCX 55 kb) [file 12906_2016_1265_MOESM2_ESM.docx]

**Effect of KBH-1 on free fatty acid-stimulated lipid deposition and cell viability**

To compare the alleviation effect of *Saururus chinensis* (SC), *Curcuma longa* (CL), *Polygala tenuifolia* (PT) and KBH-1 extracts on lipid accumulation of HepG2 cells, the intracellular triglyceride was quantified using adipored assay kit. In this result, KBH-1 extracts enhanced the inhibition effect of lipid deposition comparison with SC, CL, and PT (Fig. S1B), but this extract did not show cytotoxicity in HepG2 cells (Fig. S1A). These results indicate that the inhibitory effect of KBH-1was not due to cytotoxicity.

**Figure S1.**

**
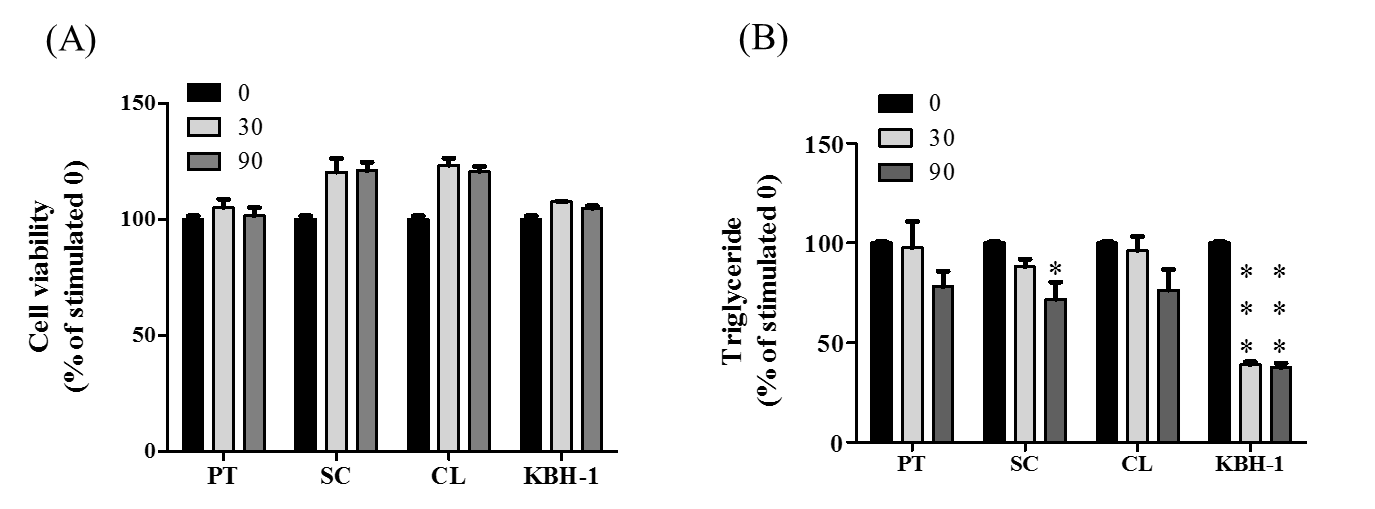
**

**Figure S1. Effect of KBH-1 on cell viability and lipid accumulation.** HepG2 cells was routinely cultured in DMEM/F12. The cells were treated with various concentration (0-90 µg/ml) of *Saururus chinensis* (SC), *Curcuma longa* (CL), *Polygala tenuifolia* (PT) or KBH-1 extracts for 6 h, then exposed to a mixture for FFA (oleic acid/palmitic acid at 2:1). (A) Intracellular triglyceride contents were examined using the AdipoRed Assasy kit. (B) Cell viability was determined by the CCK assay. The results were confirmed by three independent experiments, which were each conducted in triplicate. Data are expressed as the mean ± SEM. Significant differences from control (0 µg/ml) are indicated by **p* < 0.05, or ****p* < 0.005.
